# Occupational exposure of cashiers to Bisphenol A via thermal paper: urinary biomonitoring study

**DOI:** 10.1007/s00420-016-1132-8

**Published:** 2016-04-28

**Authors:** Sophie Ndaw, Aurélie Remy, Danièle Jargot, Alain Robert

**Affiliations:** Département Toxicologie et Biométrologie, Institut National de Recherche et de Sécurité (INRS), Rue du Morvan, CS 60027, 54519 Vandoeuvre Cedex, France; Département Métrologie des Polluants, Institut National de Recherche et de Sécurité (INRS), Rue du Morvan, CS 60027, 54519 Vandoeuvre Cedex, France

**Keywords:** Bisphenol A, Thermal paper, Cashiers, Exposure, Biomonitoring, Urine

## Abstract

**Purpose:**

As an essential component of polycarbonate plastics and epoxy resins, Bisphenol A (BPA) is found in numerous industrial and consumer products. BPA may cause adverse health effects because of its endocrine activity. General population exposure to this compound mainly through diet is well documented. Thermal paper was also identified as a source of BPA through dermal intake. In this study, we investigated whether frequent contact with thermal paper is associated with an increase in urinary BPA excretion.

**Methods:**

We evaluated the exposure to BPA in cashiers and in non-occupationally exposed workers from several workplaces. Urinary BPA was quantified in free (unconjugated) and total (unconjugated plus conjugated) forms in 24-h and spot urine samples using LC–MS/MS. BPA concentration in thermal paper was also measured from each workplace. In addition, participants provided information on job, food and drink, tobacco consumption and hands wash during the sampling period through a questionnaire.

**Results:**

Urine samples were collected from 90 cashiers and 44 controls. Free and total BPA were detected in all samples. The median urinary total BPA concentration was 3.54 µg/L (2.89 µg/g creatinine) for controls and 8.92 µg/L (6.76 µg/g creatinine) for cashiers. For the free BPA, the median urinary concentration was 0.20 µg/L (0.21 µg/g creatinine) for controls and 0.28 µg/L (0.22 µg/g creatinine) for cashiers. Any correlation was found between the urinary concentration levels and the number of thermal receipts handled. Hand washes frequency, age, job length of service and tobacco consumption had also no effect on the BPA excretions.

**Conclusion:**

A significant increase in urinary total BPA concentration was observed for cashiers handling daily thermal paper receipts. However, no significant increase was observed in urinary free BPA concentration. These findings are particularly interesting for risk assessment since all available data on occupational exposure to BPA through thermal paper were obtained from models or from simulated experiments.

## Introduction

Bisphenol A or BPA (4,4′-dihydroxy-2,2-diphenylpropane, CAS 80-05-7) has been used in its monomer form for more than 50 years in polycarbonate plastic and epoxy resin production. World production of BPA amounts to several million tonnes/year (Vandenberg et al. 2010), making BPA one of the most widely used chemicals. Polycarbonates are used in various sectors of industry and enter into the composition of very many day-to-day objects (food containers, CDs, spectacles, etc.). Epoxy resins are used in various industrial products such as paints, adhesives, floor coverings or food tin coatings and drink cans. BPA also plays a part in synthesizing other polymers and resins (polyester, polysulfone, vinylester resins, etc.).

BPA is used as a developer in thermal papers, which include a variety of uses including cash register and credit card receipts, self-adhesive labels, etc.

Data provided by experiments on animals suggest that BPA could represent a risk for human and animal health because of its oestrogenic properties (Vom Saal and Hughes 2005; CERHR 2008). It has also been demonstrated that BPA interacts with other endocrine receptors, such as thyroid hormone receptors, for example (Diamanti-Kandarakis et al. 2009). Exposure to BPA would increase the risks of breast cancer, obesity, diabetes as well as disorders of the nervous and reproductive systems. It has also been suggested that exposure to BPA during uterine and infantile development would be a major factor contributing to the occurrence of infertility, genital tract malformations, attention deficit and hyperactivity (Vandenberg et al. 2010; Mattison et al. 2014). The toxicological effects of BPA are however controversial. In its opinion of 2015, the EFSA (2015a) performed an update of the hazard characterisation of BPA based on data from 2010 to 2012. The expert panel of EFSA concluded that toxicological studies did not provide sufficient evidence of metabolic, neurological, cardiovascular and reproductive effects of BPA. The EFSA however agreed with the French Agency for Food, Environmental, and Occupational Health and Safety (ANSES) about the adverse effects of BPA on mammary gland (ANSES 2013).

Vandenberg et al. (2007) have suggested that monomer form BPA released from consumer products would cause contamination of foods, drinking water, dust and air, and would thus lead to large scale population exposures. Diet is therefore considered to be the predominant source of BPA for humans (Geens et al. 2012a). Several biomonitoring studies have documented the exposure of general population to BPA (Pirard et al. 2012; Völkel et al. 2011; Zhang et al. 2011; Ye et al. 2008). Urinary concentration of total BPA (free plus conjugated) has most commonly been used to evaluate exposure to BPA via oral and non-oral routes. Calafat et al. (2005) revealed the presence of BPA metabolites in over 90 % of urine samples from a cohort representing the American population (children and adults), with a median concentration of 1.28 µg/L. A recent review of urinary BPA concentration in general population is available from Geens et al. (Geens et al. 2012a).

However, during these last years, exposure to BPA during handling of thermal papers is of increased interest. These papers are coated on one of their faces with a layer of BPA, which under the action of a heating head, allows printing by reacting with the solvent and the ink on the paper. In these thermal papers, BPA is in a non-polymerised form which can be transferred to the skin during contact with the paper. Experiments using human skins have shown that BPA is absorbed by the skin following direct contact with its monomer form (Demierre et al. 2012; Marquet et al. 2011). BPA concentration in thermal paper is usually of the order of 1–2 mg per 100 mg of paper (Geens et al. 2012b). The quantity of BPA transferred to the finger after handling thermal paper has been estimated by Biederman et al. (2010) to 1.1 µg and this quantity could increase by a factor of 10, when in contact with wet skin. Handling thermal paper could contribute to exposure to BPA through dermal but also orally when there is a contact between mouth and unwashed hands. A few studies have been focused so far to this topic, especially regarding cashiers who handle thermal paper daily. Braun et al. (2011) had found that the excreted levels were slightly higher in cashiers (geometric mean 2.8 µg/g of creatinine, *n* = 17) than in unemployed women (geometric mean 1.9 µg/g of creatinine, *n* = 82). However, no data on the presence of BPA in paper handled by these workers was available. Ehrlich et al. (2014) followed 12 volunteers handling thermal receipt paper for 2 h in a simulation study. In this case, the mean urinary BPA concentration was higher after handling the receipts or tickets. More recently, in a study conducted by Porras et al. (2014), three volunteers simulated the work of cashiers in a supermarket. The BPA concentration excreted after simulation did not exceed the limit value, which corresponds to the 95th percentile of the control group.

The aim of this study was to investigate whether frequent contact with thermal paper is associated with an increase in urinary BPA excretion. We evaluated the exposure to BPA in cashiers and in non-occupationally exposed workers and from several workplaces. Urinary BPA was quantified in free (unconjugated) and total (unconjugated plus conjugated) forms. BPA concentration in thermal paper was also measured from each workplace.

## Materials and methods

### Chemicals

BPA (>99 %) and the internal standard ^2^H_6_-BPA (98 %) were supplied by Sigma-Aldrich. Dansyl chloride (DNS) used as derivatisation reagent, β-glucuronidase (Helix pomatia type HP2), BPA-monoglucuronide (>96 %) which was used to evaluate the efficacy of deconjugation, sodium acetate, sodium carbonate, sodium bicarbonate and toluene (chromasolv plus) were also purchased from Sigma-Aldrich. Formic acid, acetic acid, methanol (LC–MS grade) and acetonitrile (LC–MS grade) were obtained from Fluka. Acetone was purchased from Carlo Erba. Ultrapure water was produced by a Direct Q system from Millipore.

### Standard solutions

Individual stock solutions of BPA and ^2^H_6_-BPA were prepared in acetonitrile at 1 g/l and stored at −20 °C. The stock solution of BPA-monoglucuronide was prepared in methanol at 1 g/l and stored at −20 °C. Working solutions were prepared by diluting the stock solution in pure water. Appropriate serial dilutions of intermediate solutions with a pool of human urine from members of our laboratory staff were prepared just before use for calibration in the range 1–100 µg/L.

### Thermal paper analysis

Approximately 100 mg of thermal paper were accurately weighed, cut into small pieces and dispersed in 40 ml of a methanol/water mixture (90:10). Extraction of BPA from thermal paper was performed by sonication for 15 min. The extract was then filtrated before analysis by high-performance liquid chromatography with fluorimetric detection (Perkin Elmer system). HPLC separation was achieved on an Alltima C18 column (150 × 3, 5 µm) from Grace. The mobile phase used was a mixture of acetonitrile/water (35:65) in isocratic mode. The flow rate of the mobile phase was 1 ml/min and the injection volume 10 µl. The fluorimetric detector operated at an excitation wavelength of 230 nm and at an emission wavelength of 315 nm. Quantification was performed by external calibration. The recovery rate of the extraction was about 98 % and the detection limit was 0.73 µg/L or 0.03 % (0.03 mg BPA/100 mg paper).

### Urine samples analysis

The protocol used in this study was adapted from the method described and published by Fox et al. (2011) with slight modifications. BPA was derivatized with dansyl chloride before analysis by high-performance liquid chromatography tandem mass spectrometry. To measure total BPA, 600 µl of a β-glucuronidase solution (prepared by mixing 100 µl of crude enzyme with 8 ml of water and 2 ml of 1 M pH 5 sodium acetate buffer) and 60 µl of internal standard (^2^H_6_-BPA, 100 µg/L) were add to 600 µl of urine sample in a glass vial. The sample solution was vortexed, incubated overnight at 37 °C and was then cooled down at room temperature. The solution (1 ml) was applied then on Extrelut cartridge (NT1, Merck) during 10 min and the analytes were eluted with 10 ml of toluene. The eluate was evaporated to dryness under a stream of nitrogen and the residue was redissolved in 200 µl of 250 mM carbonate buffer (pH 9.0). Derivatization was performed with 200 µl of 1 g/l solution of dansyl chloride in acetone and the sample was heated at 65 °C for 30 min. For the determination of free BPA, the internal standard was added to the urine sample and the sample was extracted, without the deconjugation step. The reagent blanks were treated as urine samples.

### Chromatographic conditions

The samples were analysed on a TSQ Quantum Ultra triple quadripole mass spectrometer from Thermo Fisher Scientific. The MS/MS system operated in positive mode with the following parameters: vaporizer temperature, 300 °C; capillary temperature, 375 °C; sheath gas (N_2_) pressure, 40 units; auxiliary gas (N_2_) pressure, 30 units; ion sweep gas (N_2_) pressure, 2 units; collision gas (Ar) pressure, 1.5 mTorr; chrom filter peak width, 5.0 s; scan width, scan time, 0.8 s; peak width, 0.2 Da Q1 and 0.7 Da Q3. Chromatographic separation was achieved on a Kinetex C_18_ (100 × 2.1 mm, 2.6 µm) from Phenomenex with a mixture of 0.1 % formic acid (solvent A) and acetonitrile (solvent B) as mobile phase. The starting eluent (60 % A) was applied for the first min. The proportion of acetonitrile was then increased to 95 % over a period of 4 min and maintained for 1 min. The mobile phase was then immediately adjusted to its initial composition and the column was equilibrated for 4 min. The flow rate of the mobile phase was 0.4 ml/min and the injection volume 5 µl. Signal acquisition was performed in SRM mode monitoring with the ion transitions: 695–170 m/z and 701–170.8 m/z for the BPA-DNS derivative and ^2^H_6_-BPA derivative respectively.

Urinary creatinine was measured in urine samples using the Jaffé colorimetric method.

### Method validation

The validation criteria of the method were assessed in pool of spiked urine samples. Linear regression analysis was used to construct calibration curves. Within-day and between-day precision and accuracy were evaluated by determining BPA in three quality control (QC) samples prepared at nominal urine concentrations of 0.2, 1, 5 µg/L in six replicates on three different days. The precision of the method at each QC concentration was expressed as a coefficient of variation (CV) by calculating the standard deviation as a percentage of the mean calculated concentration, while the accuracy of the procedure was determined by expressing the mean calculated concentration as a percentage of the added concentration.

The limit of quantification (LOQ) was estimated by analyzing blank urine samples spiked with ^2^H_6_-BPA internal standard. LOQ is defined as the lowest concentration which can be quantified with accuracy within 20 % of the nominal value and a precision which should not exceed 20 %. To assess the reliability of the overall method, relative matrix effect was evaluated by determining the precision of standard line slopes (expressed as CV %) in three different of urine lots, as previously described (Matuszewski 2006). The enzymatic conversion of the glucuronide conjugate to BPA by β-glucuronidase was monitored by submitting three replicates of urine samples fortified with 100 and 200 µg/L of BPA-monoglucuronide to the overall method. Calculation of the percent conversion of BPA-monoglucuronide to BPA was based on the theoretical concentration of BPA that would result from complete conversion.

### Field study

This study took place between July 2013 and June 2014. Thermal paper receipts were collected from several workplaces to identify those where BPA containing thermal papers were handled. Ten companies, including restaurants, bookshop, hairdressers, garden centre, ticket office, shops, do-it-yourself store and hotel were recruited to take part in this study. The potentially exposed employees at these workplaces were grouped under the generic term “cashiers” and handled a more or less large number of receipts every working day. A total of 90 cashiers were recruited for urinary BPA monitoring. We also investigated the urinary BPA levels of 44 non occupationally exposed workers as a control group. They were recruited in the same companies whenever possible and their job did not involve any occupational exposure to BPA. The controls were from administrative staff, IT department or supply staff.

Urine samples over a 24 h period (24-h urine samples) or spot urine samples, including pre-shift and post-shift samples and first morning void on the following day were collected from each participant, during 1 or 2 days. Urines samples were stored after collection at −20 °C until analysis.

Urine collection was accompanied by a questionnaire providing information on job, food and drink, tobacco consumption, hands wash, adhesives and paints uses during the sampling period. The number of receipts handled by cashiers was estimated by analyzing the recorded transactions. Furthermore, a second set of thermal paper receipts was collected during the urine sampling period from the selected workplaces to confirm BPA presence in the thermal paper and ensure that any substitution of BPA has not occurred.

All the participants were recruited on a voluntary basis and gave their written consent for this investigation. The study received the authorisation of the Agence Nationale de Sécurité du Médicament et des produits de santé (ANSM) [French national drug and health product safety agency] and was approved by the local ethic committee Comité de Protection des Personnes Est III (CPP) [personal protection agency for Eastern France].

### Statistical analyses

Descriptive and inferential statistical analyses were performed using Stata 13.0 software. The mixed linear regression model was used to test the effects of different variables on urinary BPA (according to its different expressions: free or total forms, adjusted or not by creatinine). This type of model allowed us to take into account data non-independence (urine samples from same subjects, themselves from the same company) by integrating a subject random effect and a company random effect. A logarithmic transformation was applied to the data on urinary BPA in its different forms.

The questionnaires were analyzed for the food and drink consumption. A Pearson’s Chi-squared test was performed to determine whether the control group and cashiers had similar food and drink habits.

The statistical significance threshold was set at 5 %.

## Results

### Method validation

The method was linear over a wide concentration range up to 100 µg/L with a determination coefficient *R*^2^ of 0.990 (mean of 5 calibration curves). Analytical QC results are reported in Table 1. The within-day precision varied from 1.1 to 9.7 % at the lower concentration while between-day precision ranged from 4.9 to 11.4 %. The method was shown to be accurate, with intra-day accuracy always higher than 88 %.

**Table 1:** Within-day and between-day precision and accuracy of the urinary BPA determination method

Nominal concentration µg/L urine, *n* = 6	Within-day precision CV (%)	Between-day precision CV (%), *n* = 3 days	Accuracy % of nominal concentration
0.2	9.7	11.4	88.2
1	1.1	10.3	102.1
5	2.3	4.9	104

The urinary limit of quantification of the assay for ^2^H_6_-BPA was found to be 20 ng/L. At this concentration, the within-day precision was 12.5 % and the accuracy 91 %.

Overall method reliability was investigated by determining relative matrix effects. The precision of the standard line slopes from three different urine lots, expressed as CV (%), did not exceed 7.7 %, indicating that the method can be considered reliable and free from relative matrix effect liability.

The enzymatic conversion of a solution of 200 µg/L of BPA-monoglucuronide to BPA by β-glucuronidase averaged 101 % with a CV of 7.8 %. All solvents and materials used for the overall method were carefully chosen to ensure that exogenous contamination of urine samples by BPA remained minimal. Background levels of BPA, determined before each run, were in the range of 60 ng/l and urinary measured concentrations were corrected by the background level.

### Study population and BPA levels in thermal receipts

The distribution of exposed and control employees in the different companies is shown in Table 2, as the range of the number of receipts handled and the BPA concentrations in those receipts. BPA concentration in thermal paper receipts ranged from 0.93 to 1.81 % which is consistent with previously published data (Liao and Kannan 2011; Geens et al. 2012b). All cashiers have handled receipts during the study. However, the number of receipts handled was highly variable among cashiers and depending on the workplace, ranging from 10 to 1000 receipts a day. A working day was set to 8 h. Cashiers from the two hairdressers (companies 7 and 8) have handled the lowest number of receipts and the highest numbers were reported by cashiers from a restaurant (company 5). Cashiers recruited at company 4 were classified afterward in the control group as there was no more BPA in the thermal paper receipts.

**Table 2:** Overview of recruited companies, range of the number of receipts handled by cashiers and BPA concentrations in receipts

Companies	Categories	Sample size		Range of receipts handled/day	% BPA (mg BPA/100 mg paper)
		Controls	Cashiers		
1	Leisure park^a^	7	14	25–494	1.17
2	Ticket office	2	12	40–290	0.93
3	Garden centre	10	–	10–210^b^	–
4	Leisure park^a^	14	40	10–450	1.70
5	Restaurant	5	8	160–1,000	1.60
6	Restaurant	2	2	150–230	1.81
7	Hairdresser		1	10	1.75
8	Hairdresser		4	10–20	1.61
9	Hardware store	1	3	20–210	0.99
10	Bookshop	3	6	50–670	0.96
Total		44	90		

^a^The leisure park consisted of restaurants, shops, hotels and ticket offices

^b^Thermal paper receipts with no BPA

There was a majority of women (69 women) among the 90 cashiers aged 20–60 years. In the control group, there were 21 women and 23 men (Table 3). The median age was 41 years in the control group. In the cashier group, the median age was 32 years and the median number of years as cashier was 6 years (Table 3).

**Table 3:** Distribution of the population studied based on sex, age and job length of service

	Sample size	Woman	Man	Age median [range]	Job length of service (years) [range]
Controls	44	21	23	41 [21–59]	9.5 [<1–22]
Cashiers	90	69	21	32 [20–60]	6 [<1–41]

### Urinary BPA levels in 24-h urine samples

**Fig. 1:**
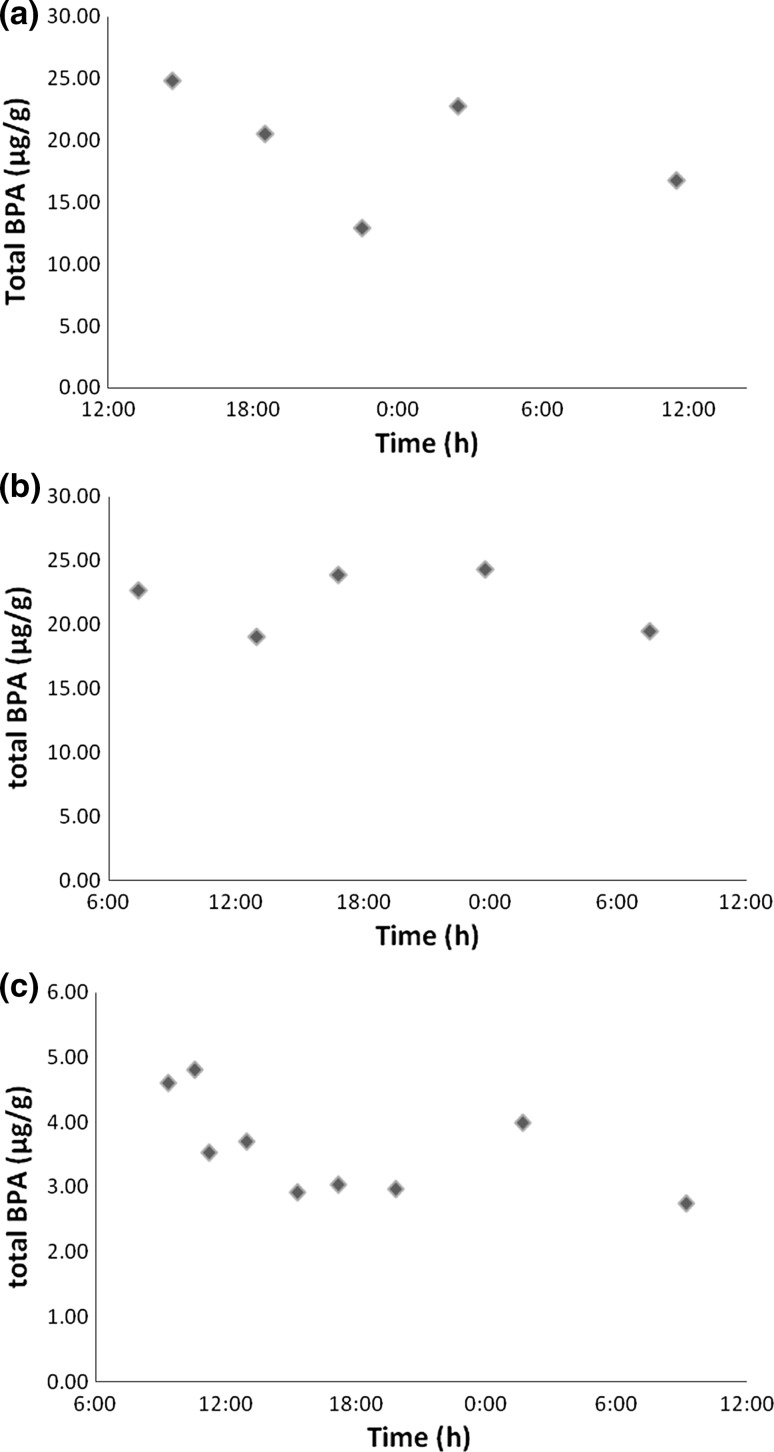
Urinary BPA concentrations (µg/g) over 24 h **a** restaurant’s cashier working hours 16:00–0:00; **b** ticket office’s cashier working hours 09:00–17:00; **c** control working hours 11:00–19:00

At company 1, 24-h urines samples were collected to assess the elimination kinetic profile of BPA and determine the best sampling strategy for an occupational exposure assessment purpose. Cashiers were from a restaurant (*n* = 7) and from a ticket office (*n* = 7). Figure 1 shows the urinary total BPA concentrations versus time for a cashier in each place and for a control over 24 h. The BPA elimination profiles for the cashiers were rather uncharacteristic, similar to the control profile and did not feature excretion peaks related to occupational exposure. The elimination profiles exhibited few variations in urinary total BPA concentration over 24 h. The urinary elimination profiles from the other cashiers in this company were not very different even if some higher urinary levels were observed occasionally. Table 4 shows the median of total BPA excreted per day by cashiers and controls (expressed in µg/24 h) and median concentrations (expressed in µg/g, creatinine) for post-shift and first morning urine samples. The median of total BPA excreted was 4.8 µg/24 h in the control group, 18.4 µg/24 h for the restaurant’s cashiers and 14.6 µg/24 h for the ticket office’s cashiers.

**Table 4:** Urinary total BPA concentrations of cashiers and controls at company 1

	Cashiers				Control (*n* = 7)	
	Restaurant (*n* = 7)		Ticket office (*n* = 7)			
	Median	Range	Median	Range	Median	Range
Total BPA/day (µg/24 h)	18.4	9.4–43.5	14.6	3.5–42.6	4.8	2.5–6.2
Total BPA in post-sift samples (µg/g)	8.0	4.8–23.0	8.0	3.2–24.0	3.0	1.8–8.0
Total BPA in first morning samples (µg/g)	12.0	1.0–17.0	9.0	3.0–19.0	3.0	2.0–7.0

We performed a Kruskal–Wallis test to determine whether the excretions varied significantly between cashiers and control. Total BPA excretions were significantly higher in the cashier group for the total mass of BPA excreted per day (*p* = 0.003, 95 % confidence level), for the BPA concentration in post-shift samples (*p* = 0.04, 95 % CI) and for the BPA concentration in first morning samples (*p* = 0.007, 95 % CI). It appears that 24-h samples, post-shift samples or first-morning void samples collection would be relevant for occupational exposure assessment in cashiers. A urine spot sample collection protocol including pre-shift and post-shift samples and first morning void was therefore subsequently applied for the study.

### Urinary BPA concentration levels

In total, 195 urine samples were collected from the control group and 390 urines samples from cashiers. Total BPA as well free BPA were detected in 100 % of samples thereby confirming the ubiquitous nature of this compound. Table 5 shows the concentration of free BPA and total BPA from the control group, with and without urinary creatinine adjustment. The median total BPA concentration for the control group was 3.54 µg/L with a range of 0.10–36.6 µg/L. For the free BPA, the concentrations ranged from 0.01 to 1.38 µg/L with a median of 0.20 µg/L.

**Table 5:** Urinary concentrations of free and total BPA (in µg/L and in µg/g) from the control group

	*n* ^a^	Min	Median	95th percentile	Max	GM (GSD)	AM
Total BPA (µg/L)	195	0.10	3.54	14.2	36.6	3.52 (2.35)	4.99
Free BPA (µg/L)	195	0.01	0.20	0.73	1.38	0.21 (2.33)	0.29
Total BPA (µg/g)^b^	181	0.44	2.89	8.69	20.8	3.0 (1.90)	3.75
Free BPA (µg/g)^b^	181	0.01	0.21	0.82	2.26	0.18 (2.72)	0.28

*GM* geometric mean, *GSD* geometric mean standard deviation, *AM* arithmetic mean

^a^Number of samples

^b^Creatinine adjusted

Geometric mean, minimum, maximum, median and 95th percentile values of urinary free and total BPA concentrations for cashiers are shown in Table 6. The median total BPA concentration for cashiers was 8.92 µg/L, the concentrations ranging from 0.54 to 1915 µg/L. The maximum total BPA concentration measured at 1915 µg/L was an exceptional urinary level considering the 95th percentile value of 44.0 µg/L. However, no explanation could be found when analyzing the questionnaire. For the free BPA, the concentrations ranged from 0.01 to 16.2 µg/L with a median of 0.28 µg/L.

**Table 6:** Urinary concentrations of free and total BPA (in µg/L and in µg/g) from the cashiers group

	*n* ^a^	Min	Median	95th percentile	Max	GM (GSD)	AM
Total BPA (µg/L)	390	0.54	8.92	44.0	1915	8.58 (2.83)	20.2
Free BPA (µg/L)	390	0.01	0.28	0.88	16.2	0.28 (2.17)	0.42
Total BPA (µg/g)^b^	352	0.68	6.76	24.8	704	7.10 (2.26)	12.0
Free BPA (µg/g)^a^	352	0.02	0.22	1.07	9.19	0.23 (2.30)	0.36

*GM* geometric mean, *GSD* geometric mean standard deviation, *AM* arithmetic mean

^a^Number of samples

^b^Creatinine adjusted

The median percentage of the free BPA form represented 6.3 % (0.3–50 %) and 3.3 % (0.3–65 %) in the control and exposed populations respectively.

Since the BPA exposure in the control group was expected to be only of environmental origin and thus independent of working hours, no distinction was made according sampling time. For cashiers, geometric mean value of BPA concentration for pre-shift, post-shift and first morning urine samples are shown in Table 7.

**Table 7:** Urinary concentrations (in µg/L) of free and total BPA in pre-shift, post-shift and first morning void samples from the cashiers group

	*n* ^a^	Total BPA (µg/L)	Free BPA (µg/L)
		GM (GSD)	GM (GSD)
Pre-shift samples	90	7.85 (3.10)	0.35 (2.55)
Post-shift samples	91	10.5 (2.77)	0.30 (2.01)
First morning void samples	87	10.1 (2.75)	0.32 (2.0)

*GM* geometric mean, *GSD* geometric mean standard deviation

^a^Number of samples

The analysis of the questionnaires by a Pearson’s Chi-squared test has shown that there was no significant difference between the cashiers and the control group for food and drink habits. We analysed subsequently free and total BPA excretions in relation with different variables using mixed regression models on the log-transformed urinary BPA data.

Concentrations of total BPA in the cashiers group turned out to be significantly greater than those in the control group, irrespective of the sampling time (*p* < 0.000 for pre-shift, post-shift and first morning urine samples). These results confirm the data previously obtained at company 1.

Concentrations of total BPA were also higher in post-shift and first morning urine samples than pre-shift samples (*p* = 0.001 and *p* = 0.014).

**Fig. 2:**
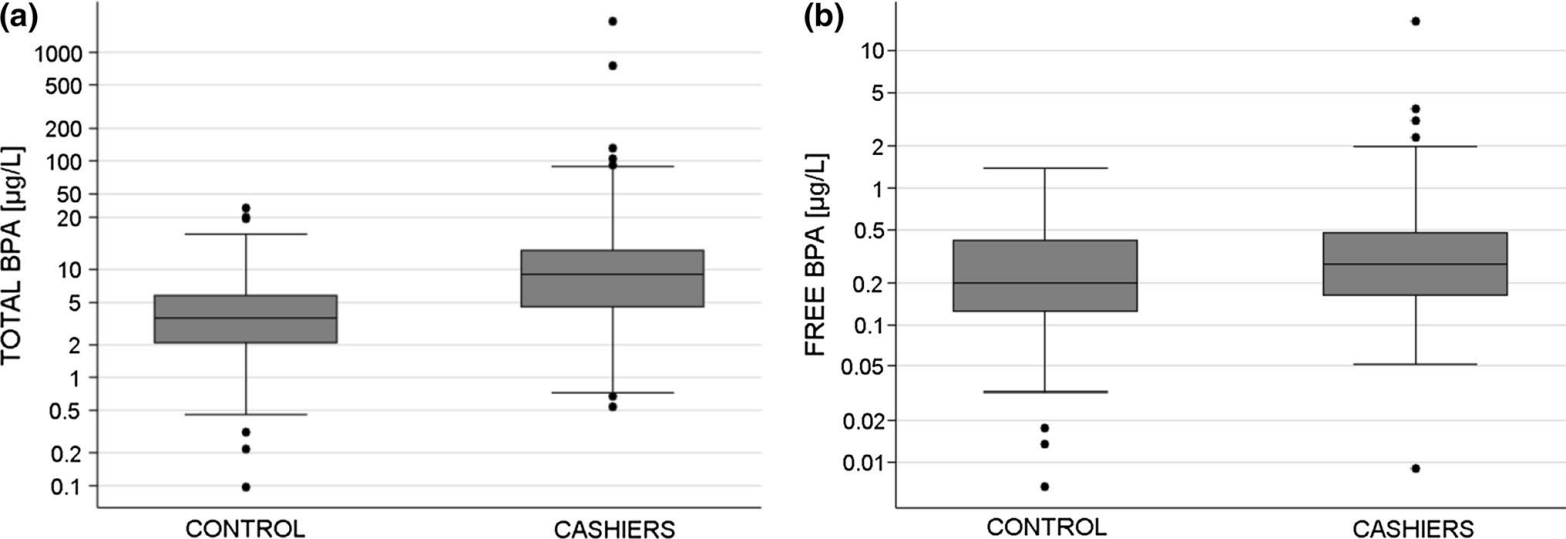
Box-plots of total BPA (**a**) and free BPA (**b**) concentrations in cashiers and controls. *p* value for total BPA concentration: *p* < 0.000

Figure 2 illustrates the urinary BPA concentration distribution in the control group and in the cashiers group expressed on a logarithmic scale. Total BPA level was significantly higher in the cashiers group (*p* < 0.000). However, for free BPA concentration, no difference was found between the two groups. Statistical analysis performed with values adjusted for creatinine lead to the same conclusions.

**Fig. 3:**
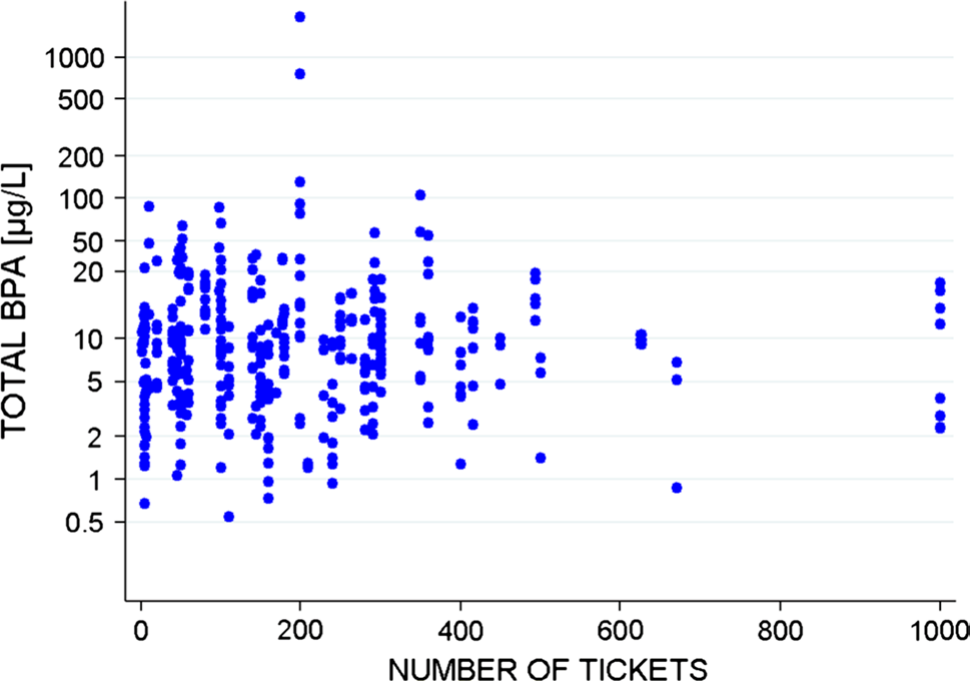
Distribution of total BPA concentrations in cashiers according to the number of receipts handled

The association between the number of receipts handled by cashiers and the urinary BPA excretion was tested. The total BPA level was not correlated with the number of receipts (Fig. 3). Hand washes frequency, age, job length of service and tobacco consumption had also no effect on the BPA excretions.

The exposure effect was also tested on the female population. Geometric means values for free and total BPA concentrations and for women in the control group were 0.19 (GSD 2.53) µg/L and 3.16 (2.46). For women in the cashiers group, geometric means values were 0.28 (2.06) µg/L and 7.89 (2.86). No significant difference between men and women was observed for BPA exposure

## Discussion

The unconjugated (or free) BPA is considered to be the biologically active form for the effects related to estrogen receptors. Therefore, the data on level of free BPA is important for the assessment of human health risks. Total BPA (free plus conjugated forms) provides information about the extent of exposure. For this reason, both free and total BPA levels are important and reported in this study. The dosage of BPA in urine, particularly the free form, is yet challenging. Given the low concentrations of free BPA in urine, reliable analytical methods that provide very low detection limits need to be developed. Besides, since BPA is ubiquitous in the environment, the suppression or minimizing of background contribution during samples collection, storage, extraction and analysis, to avoid any exogenous contamination became crucial (Markham et al. 2010). And finally, the main BPA conjugated form (BPA-monoglucuronide or BPAG) has been shown to be unstable under certain conditions (Ye et al. 2007). Hydrolysis of BPAG in BPA during sample conservation and processing must be avoided during the determination of free BPA.

In this study, the urine samples were thus obtained under controlled conditions by our laboratory team in each company and rapidly frozen at −20 °C after collection until analysis. The stability of BPA forms in urine was checked. The free BPA remains stable and the conjugated forms were not hydrolyzed to BPA up to 24 h at room temperature (data not shown). However, we made sure that urine samples were kept the shortest time possible at room temperature. To minimize the BPA background contribution, all solvents, vials, containers, pipettes were checked for BPA contamination. Low BPA-containing solvents were chosen and glass vials and pipettes were used. The remaining background contamination was monitored before each run and all reported results were corrected by the background level (Kubwabo et al. 2014). Due to the background contamination, the limit of quantification was estimated using spiked urine samples with a surrogate ^2^H_6_-BPA (Markham et al. 2010; Buscher et al. 2015). The measurement of BPA by LC–MS/MS as dansyl derivative has allowed the development of a very sensitive method. However the limit of quantification of 20 ng/l is just informative for the method performance since the blank value for BPA was calculated to be 60 ng/l. The background contamination could lead to a lesser accuracy and more variability, particularly for low concentrations. Nevertheless, the validation data showed acceptable accuracy and precision in human urine for the lowest QC concentration.

### Total BPA levels in the control group and in the general population

Several biomonitoring studies have been conducted worldwide and have documented the large scale exposure of general population to BPA (Vandenberg et al. 2010; Geens et al. 2012a). The data reported in the literature, expressed in total BPA, exhibit widespread disparity within the study populations with geometric means between 1 and 4 µg/L for an adult population (Geens et al. 2012a ). The geometric mean is 1.29 µg/L for the Canadian population (Haines and Murray 2012), 1.79 µg/L for the American population (CDC 2014) and 1.55 µg/L for the German population (UBA 2012). The geometric mean of the total urinary BPA concentration of the control group (*n* = 44) in this study of 3.52 µg/L was higher than the aforementioned populations but in the same order of magnitude. Unfortunately, no data for the French general population are currently available to ensure that our controls, which were from the same companies as cashiers, were representative of the French general population.

It is generally recognized that diet is the predominant source of BPA exposure for humans. As the objective of this study was to investigate whether frequent contact with thermal paper is associated with an increase in urinary BPA excretion, attention was required to avoid bias from food and drink habits. Detailed information were collected through a questionnaire on diet but also smoking and do-it-yourself habits. As there was no difference between our control group and cashiers concerning these factors, it was relevant to compare the cashiers to our non-occupationally exposed group for BPA excretion.

### Variability of urinary BPA concentrations

Several authors have reported the variability of the urinary BPA concentrations over time due to the different exposure sources and the rapid urinary BPA elimination. This variability is independent of the sampling strategies (spot urine samples, 24-h urine samples) and is observed between-day and between-person but also within-day and within-person (Lassen et al. 2013; Ye et al. 2011). Therefore, we collected three spot urine samples including first morning void from each participant and for a daily basis to strength our biomonitoring data (Geens et al. 2012a). Furthermore, the first and the second urine spot samples (pre-shift and post-shift samples) were relative to the work shift and not to the meals. In addition, the fairly large population recruited in this study has allowed us to collect a significant number of urine samples to determine the mean excretion levels.

### Urinary elimination of BPA

The urinary total BPA elimination profile for cashiers highlighted in this study i.e. slight variations of BPA concentrations throughout the day may suggest a continuous exposure during and after the work shift. Especially as the BPA concentrations remains higher than in the control group even after the end of thermal paper handling as shown by data from first morning urine samples. In their study about in vivo and ex vivo percutaneous absorption of BPA in rats, Marquet et al. (2011) have shown that the BPA present in the skin at the end of exposure is available for diffusion and absorption. Thus the skin may constitute a reservoir for BPA which could explain the higher urinary half-life for dermal route compared to i.v. route (28 and 10 h respectively). In a previous study, Biedermann et al. (2010) had shown that no increase in the quantity of BPA transferred to a finger was observed following repeated contact, which indicates a probable equilibrium between the BPA quantity on the paper and on the skin surface. Given our kinetic data, it may be assumed that, in addition to a saturation phenomenon, BPA may be stored in the skin and be subject to constant absorption. Under these circumstances, one of the questions raised is how much time is required after exposure for a total washout? Unfortunately, we have not been able to investigate this issue. It is admitted that five urinary half-life are required for total washout. Using the data from rats experiments (Marquet et al. 2011), this could represents 140 h or almost 6 days.

### Urinary free BPA levels in the control group and in the general population

Studies based on animal models suggest that free BPA represents <3 % of total BPA (Völkel et al. 2002; Mattison et al. 2014). As free BPA would be the active form, its concentration could be considered a relevant indicator of potential BPA effects, at least for the effects related to estrogen receptors. However, few biosurveillance studies provide results expressed in free BPA, probably because of the need for a very sensitive dosage method and optimum conservation of urine samples prior to analysis (Ye et al. 2005; Calafat et al. 2008; Schoringhumer and Cichna-Markl 2007). In our study, free BPA was detected in all samples. In the study conducted by Liao and Kannan (2012) the proportions of free BPA and its conjugated forms was determined in 31 non-occupationally exposed persons. The mean percentage of free BPA (geometric mean 0.70 µg/L) was 32 % of the total BPA (geometric mean 5.40 µg/L). Provencher et al. (2014) reported a percentage of 1.7 % for free BPA (median concentration 0.012 µg/L) from urine samples of 46 volunteers. Kubwabo et al. (2014) measured BPA concentrations in urine samples from 36 pregnant women, and free BPA was detected in 22 % of samples (median 0.185 µg/L). The concentration of free BPA in our control group was 0.20 µg/L and appeared to be significantly lower than in Liao and Kannan (2012) study and consistent with Kubwabo et al. (2014) data. The unconjugated BPA represented 6.3 % of the total BPA concentration in controls.

The report of unconjugated BPA concentration is often controversial and questioned in the literature. In recent studies on pharmacokinetics of BPA in humans following an oral administration, percentages of unconjugated BPA in urine below 1 % of the total BPA were reported (Thayer et al. 2015; Teeguarden et al. 2015) which would be due to an extensive first pass metabolism in liver. These authors came to the conclusion that urine samples containing free BPA above 1 % were unlikely and should be considered contaminated during sample processing. Thayer et al. (2015) recognized also that it is challenging to extrapolate this conclusion to samples collected in general population because exposure is likely not exclusively by ingestion. In case of dermal exposure for example, the first pass metabolism would not occur thus leading to higher concentrations of free BPA.

### Exposure of cashiers to BPA

We observed in our study a significant increase in total BPA urinary concentration in cashiers when compared with a control group. The median concentration for total BPA was 8.92 µg/L, approximately 2 to 3 higher than the median concentration of 3.52 µg/L in the non-occupationally exposed group. To our knowledge, this is the first reporting level of BPA in urine of cashiers under real working conditions. Dermal intake of BPA after handling thermal paper has been modelled by Biedermann et al. (2010) and a potential exposure of 71 µg/day was estimated when handling frequently thermal paper during 10 h. This dermal exposure could result to a urinary BPA concentration of 40 µg/L with human skin absorption of 27 % (Porras et al. 2014; Krishnan et al. 2010).

In two recent studies, simulation experiments of cashier work were carried out. In the first study, Ehrlich et al. (2014) followed 23 participants who printed and handled thermal paper receipts continuously for two hours. The geometric mean urinary BPA concentration was 1.8 µg/L before simulation and 5.8 µg/L after simulation, clearly indicated a significant increase in BPA concentration after handling receipts. No significant increase was observed when using gloves. In the second study, Porras et al. (2014) conducted two experiments with three volunteers. In experiment 1, the volunteers handled paper receipts about 140 times during a working day of 8 h. In experiment 2, the volunteers handled receipts during 5 min with their hands previously rubbed by a hand cream and the operation was repeated 2 times. The authors set the 95th percentile of BPA urinary concentration (8 µg/L) of a non-occupationally exposed population (*n* = 121) as the reference limit value. All urinary BPA measurements in experiment 1 and 2 remained below 8 µg/L. Although Porras et al. (2014) study was conducted in carefully controlled experimental conditions, it obviously suffers from a lack of statistical capacity.

The strength of our study is that we have recruited a significant population of cashiers from different companies, handling a variable amount of thermal paper receipts during the working day, thus with an exposure level variable. Our population was rather representative of this occupational group.

Since the first pass metabolism in liver would not occur when exposure is through dermal route, a significant increase of the unconjugated BPA concentration in cashiers was theoretically expected. Even if a slight increase of the unconjugated BPA concentration from 0.20 µg/L in controls to 0.28 µg/L in cashiers was observed, this difference was not significant in this study. Nonetheless, in a study (under publication) in an industrial printing company where thermal paper containing BPA was handled, and where exposure through inhalation could not be excluded, we found a significant difference between controls and printing workers.

The absence of a relation between the number of receipts handled and the total BPA concentration found in our study supports the hypothesis of Biedermann et al. (2010) about an equilibrium phenomenon between the BPA quantity on the paper and on the skin surface, suggesting that cashiers have a quite constant amount of BPA on the skin during their working day. Moreover, Marquet et al. (2011) have shown that there was no accumulation of BPA in the skin throughout exposure in their experiment. The type of receipts or the contact time could be another explanation for the absence of relation between the number of receipts handled and the total BPA concentration. The receipts were handled by cashiers throughout the working day and the contact time could not be controlled because our study was conducted under real working conditions. Furthermore, the receipts handled in this study were similar and differed only by the amount of BPA in thermal paper. And the urinary BPA level was not correlated with the amount of BPA in thermal paper (data not shown).

We have shown in this work that frequent contact with thermal paper is associated with an increase in urinary BPA excretion for cashiers. The urinary levels found in cashiers are the result of both environmental and occupational exposure. Environmental exposure can lead in some cases to a significant contamination which can result to a misinterpretation of occupational data. However, when compared to a control group, as in this study, which is similar to the cashiers group except for the occupational part, the role of thermal paper handling on the increase in urinary total BPA concentration is quite clear.

The non-dietary sources (dust, cosmetics, thermal paper) of BPA were considered in the EFSA opinion of 2015. Average dermal exposure through thermal paper of 58.9 ng/kg bw per day was estimated for the adult population. By applying an absorption fraction of 10 %, the internal dose from dermal exposure would be 5.9 ng/kg bw per day (EFSA 2015b). Thermal paper was assumed to be handled in this case once a day by the fingertips of three fingers of one hand. The expert panel of EFSA concluded that there was no health concern from dietary or aggregated (from diet, dust, cosmetics and thermal paper) exposure when compared with the temporary Tolerable Daily Intake (t-TDI) of 4 µg/kg bw per day. However, occupational exposures to BPA were not included in this opinion. On the other hand, the French experts of ANSES have published recently an extensive risk assessment on BPA with a focus on dermal exposure through thermal paper. The exposure resulting from thermal paper was estimated from an exposure model using an absorption rate determination and is reported as internal exposure. The 95th percentiles used in the risk assessment were 89 ng/kg bw per day and 460 ng/kg bw per day for adults in general population and a population of workers, respectively. It would be interesting to determine to what extent these estimated internal values are consistent with our biomonitoring data.

## Conclusion

To our knowledge, this is the first published data on the urinary free and total BPA concentrations among cashiers. According to our results, handling thermal paper receipts containing BPA during a working day is associated with an increase in total BPA level in urine when compare to a control group. These findings are particularly interesting for risk assessment since all available data on occupational exposure to BPA through thermal paper were obtained from models or from simulated experiments.

Although this work was focused to BPA in thermal paper, Bisphenol S (BPS) has been identified in some thermal receipts as BPA substitute. Limited studies have revealed some similar toxicological profiles for BPA and BPS. Studies on percutaneous absorption of BPS and biomonitoring data on BPS concentration in cashiers would certainly be relevant.

## References

[CR1] ANSES—French Agency for Food, Environmental and Occupational Health & Safety (2013) Evaluation des risques du bisphénol A (BPA) pour la santé humaine. Rapport d’expertise collective. Paris. http://www.anses.fr

[CR2] Biederman S, Tschdin P, Grob K (2010). Transfer of bisphenol A from thermal printer paper to the skin. Anal Bioanal Chem.

[CR3] Braun JM, Kalkbrenner AE, Calafat AM, Bernert JT, Ye X, Silva MJ, Barr DB, Sathyanarayana S, Lanphear BP (2011). Variability and predictors of urinary bisphenol A concentrations during pregnancy. Environ Health Perspect.

[CR4] Buscher B, van de Lagemaat D, Gries W, Beyer D, Markham DA, Budinsky RA, Dimond SS, Nath RV, Snyder SA, Hentges SG (2015). Quantitative analysis of unconjugated and total bisphenol A in human urine using solid-phase extraction and UPLC–MS/MS: method implementation, method qualification and troubleshooting. J Chromatogr B.

[CR5] Calafat AM, Kuklenyik Z, Reidy JA, Caudill SP, Ekong J, Needham LL (2005). Urinary concentrations of bisphenol A and 4-nonylphenol in a human reference population. Environ Health Perspect.

[CR6] Calafat AM, Ye X, Wong LY, Reidy JA, Needham LL (2008). Exposure of the U.S. population to bisphenol A and 4-tertiary-octylphenol: 2003–2004. Environ Health Perspect.

[CR7] Center for Disease Control and Prevention (2014). Fourth National report on human exposure to environmental chemicals.

[CR8] CERHR (2008) NTP-CERHR monograph on the potential human reproductive and developmental effects of bisphenol A. NIP publication No. 08-5994 19407859

[CR9] Demierre AL, Peter R, Orbeli A, Bourqui-Pittet M (2012). Dermal penetration of bisphenol A in human skin. Toxicol Lett.

[CR10] Diamanti-Kandarakis E, Bourguignon JP, Giudice LC, Hauser R, Prins GS, Soto AM, Zoeller RT, Gore AC (2009). Endocrine-disrupting chemicals: an endocrine society scientific statement. Endocr Rev.

[CR11] EFSA—European Food Safety Authority (2015). Scientific opinion on the risks to public health related to the presence of bisphenol A (BPA) in foodstuffs: part I—exposure assessment. EFSA J.

[CR12] EFSA—European Food Safety Authority (2015). Scientific opinion on the risks to public health related to the presence of bisphenol A (BPA) in foodstuffs: part II—toxicological assessment and risk characterization. EFSA J.

[CR13] Ehrlich S, Calafat AM, Humblet O, Smith T, Hauser R (2014). Handling of thermal receipts as a source of exposure to bisphenol A. JAMA.

[CR14] Fox SD, Falk RT, Veenstra TD, Issaq HJ (2011). Quantitation of free and total bisphenol A in human urine using liquid chromatography-tandem mass spectrometry. J Sep Sci.

[CR15] Geens T, Aerts D, Berthot C, Bourguignon JP, Goeyens L, Lecomte P, Maghuin-Rogister G, Pironnet AM, Pussemier L, Scippo ML, Van Loco J, Covaci A (2012). A review of dietary and non-dietary exposure to bisphenol A. Food Chem Toxicol.

[CR16] Geens T, Goeyens L, Kannan K, Neels H, Covaci A (2012). Levels of bisphenol A in thermal paper receipts from Belgium and estimation of human exposure. Sci Total Environ.

[CR17] Haines DA, Murray J (2012). Human biomonitoring of environmental chemicals—early results of the 2007–2009 canadian health measures survey for males and females. Int J Hyg Environ Health.

[CR18] Krishnan K, Gagne M, Nong A, Aylward LL, Hays SM (2010). Biomonitoring equivalents for bisphenol A (BPA). Regul Toxicol Pharmacol.

[CR19] Kubwabo C, Kosarac I, Lalonde K, Foster WG (2014). Quantitative determination of free and total bisphenol A in human urine using labeled BPA glucuronide and isotope dilution mass spectrometry. Anal Bioanal Chem.

[CR20] Lassen TH, Frederiksen H, Jensen TK, Petersen JH, Main KM, Skakkebæk NE, Jørgensen N, Kranich SK, Andersson AM (2013). Temporal variability in urinary excretion of bisphenol A and seven other phenol in spot, morning, and 24-h urine samples. Environ Res.

[CR21] Liao C, Kannan K (2011). Widespread occurrence of bisphenol A in paper and paper products: implications for human exposure. Environ Sci Technol.

[CR22] Liao C, Kannan K (2012). Determination of free and conjugated forms of bisphenol A in human urine and serum by liquid chromatography tandem mass spectrometry. Environ Sci Technol.

[CR23] Markham DA, Waechter JM, Wimber M, Rao N, Connolly P, Chuang JC, Hentges S, Shiotsuka RN, Dimond S, Chappelle AH (2010). Development of a method for the determination of bisphenol A at trace concentrations in human blood and urine and elucidation of factors influencing method accuracy and sensitivity. J Anal Toxicol.

[CR24] Marquet F, Payan JP, Beydon D, Wathier L, Granclaude MC, Ferrari E (2011). In vivo and ex vivo percutaneous absorption of [14C]-bisphenol A in rats: a possible extrapolation to human absorption?. Arch Toxicol.

[CR25] Mattison DR, Karyakina N, Goodman M, Lakind JS (2014). Pharmaco- and toxicokinetics of selected exogenous and endogenous estrogens: a review of the data and identification of knowledge gaps. Crit Rev Toxicol.

[CR26] Matuszewski BK (2006). Standard line slopes as a measure of a relative matrix effect in quantitative HPLC-MS bioanalysis. J Chromatogr B.

[CR27] Pirard C, Sagot C, Deville M, Dubois N, Charlier C (2012). Urinary levels of bisphenol A, triclosan and 4-nonylphenol in a general Belgian population. Environ Int.

[CR28] Porras SP, Heinala M, Santonen T (2014). Bisphenol A exposure via thermal paper receipts. Toxicol Lett.

[CR29] Provencher G, Bérubé R, Dumas P, Bienvenu JF, Gaudreau E, Bélanger P, Ayotte P (2014). Determination of bisphenol A, triclosan and their metabolites in human urine using isotope-dilution liquid chromatography–tandem mass spectrometry. J Chromatogr A.

[CR30] Schoringhumer K, Cichna-Markl M (2007). Sample clean-up with sol-gel enzyme and immunoaffinity columns for the determination of bisphenol A in urine. J Chromatogr B.

[CR31] Teeguarden JG, Twaddle N, Churchwell MI, Yang X, Fisher JW, Seryak LM, Doerge DR (2015). 24-hour human urine and serum profiles of bisphenol A: evidence against sublingual absorption. Toxicol Appl Pharmacol.

[CR32] Thayer KA, Doerge DR, Hunt D, Schurman SH, Twaddle NC, Churchwell MI, Garantziotis S, Kissling GE, Easterling MR, Bucher JR, Birnbaum LS (2015). Pharmacokinetics of bisphenol A in humans following a single oral administration. Environ Int.

[CR33] UBA Umweltbundesamtes (2012) Substance monograph on bisphenol A (BPA)—reference and human biomonitoring (HBM) values for BPA in urine. Opinion of the human biomonitoring commission of the german federal environment agency. Bundesgesundheitsblatt-Gesund 55:1215–1231 10.1007/s00103-012-1525-0 22936490

[CR34] Vandenberg LN, Hauser R, Marcus M, Olea N, Welshons VW (2007). Human exposure to bisphenol A (BPA). Reprod Toxicol.

[CR35] Vandenberg LN, Chahoud I, Heindel JJ, Padmanabhan V, Paumgartten JR, Schoenfelder G (2010). Urinary, circulating, an tissue biomonitoring studies indicate widespread exposure to bisphenol A. Environ Health Perspect.

[CR36] Völkel W, Colnot T, Csanady GA, Filser JG, Dekant W (2002). Metabolism and kinetics of bisphenol A in humans at low dose following oral administration. Chem Res Toxicol.

[CR37] Völkel W, Kiranoglu M, Fromme H (2011). Determination of free and total bisphenol A in urine of infants. Environ Res.

[CR38] Vom Saal F, Hughes C (2005). An extensive new literature concerning low-dose effects of bisphenol A shows the need for a new risk assessment. Environ Health Perspect.

[CR100] Ye X, Bishop AM, Reidy JA, Needham LL, Calafat AM (2007). Temporal stability of the conjugated species of bisphenol A, parabens, and other environmental phenols in human urine. J Expo Sci Environ Epidemiol.

[CR39] Ye X, Kuklenyik Z, Needham LL, Calafat AM (2005). Quantification of urinary conjugates of bisphenol A, 2,5-dichlorophenol, and 2-hydroxy-4-methoxybenzophenone in humans by online solid phase extraction-high performance liquid chromatography-tandem mass spectrometry. Anal Bioanal Chem.

[CR40] Ye X, Pierik FH, Hauser R, Duty S, Angerer J, Park MM, Burdorf A, Hofman A, Jaddoe VW, Mackenbach JP, Steegers EA, Tiemeier H, Longnecker MP (2008). Urinary metabolite concentrations of organophosphorous pesticides, bisphenol A, and phthalates among pregnant women in Rotterdam, the Netherlands: the Generation R study. Environ Res.

[CR41] Ye X, Wong LY, Bishop AM, Calafat AM (2011). Variability of urinary concentrations of bisphenol A in spot samples, first morning void, and 24-hour collections. Environ Health Perspect.

[CR42] Zhang Z, Alomirah H, Cho HS, Li YF, Liao C, Minh TB, Mohd MA, Nakata H, Ren N, Kannan K (2011). Urinary bisphenol A concentrations and their implications for human exposure in several Asian countries. Environ Sci Technol.

